# Personalized Respiratory Support in ARDS: A Physiology-to-Bedside Review

**DOI:** 10.3390/jcm12134176

**Published:** 2023-06-21

**Authors:** Salvatore Lucio Cutuli, Domenico Luca Grieco, Teresa Michi, Melania Cesarano, Tommaso Rosà, Gabriele Pintaudi, Luca Salvatore Menga, Ersilia Ruggiero, Valentina Giammatteo, Giuseppe Bello, Gennaro De Pascale, Massimo Antonelli

**Affiliations:** 1 Department of Emergency, Intensive Care Medicine and Anesthesia, Fondazione Policlinico Universitario Agostino Gemelli IRCCS, 00168 Rome, Italyteresa.michi@policlinicogemelli.it (T.M.); melania.cesarano@policlinicogemelli.it (M.C.); tommasoros22@gmail.com (T.R.); gabriele.pintaudi@policlinicogemelli.it (G.P.); luca.salvatore.menga@gmail.com (L.S.M.); ersilia4@gmail.com (E.R.); giammatteo.valentina@gmail.com (V.G.); giuseppe.bello@policlinicogemelli.it (G.B.); massimo.antonelli@policlinicogemelli.it (M.A.); 2 Istituto di Anestesiologia e Rianimazione, Università Cattolica del Sacro Cuore, 00168 Rome, Italy

**Keywords:** ARDS, AHRF, mechanical ventilation, CPAP, HFOT, NIV

## Abstract

Acute respiratory distress syndrome (ARDS) is a leading cause of disability and mortality worldwide, and while no specific etiologic interventions have been shown to improve outcomes, noninvasive and invasive respiratory support strategies are life-saving interventions that allow time for lung recovery. However, the inappropriate management of these strategies, which neglects the unique features of respiratory, lung, and chest wall mechanics may result in disease progression, such as patient self-inflicted lung injury during spontaneous breathing or by ventilator-induced lung injury during invasive mechanical ventilation. ARDS characteristics are highly heterogeneous; therefore, a physiology-based approach is strongly advocated to titrate the delivery and management of respiratory support strategies to match patient characteristics and needs to limit ARDS progression. Several tools have been implemented in clinical practice to aid the clinician in identifying the ARDS sub-phenotypes based on physiological peculiarities (inspiratory effort, respiratory mechanics, and recruitability), thus allowing for the appropriate application of personalized supportive care. In this narrative review, we provide an overview of noninvasive and invasive respiratory support strategies, as well as discuss how identifying ARDS sub-phenotypes in daily practice can help clinicians to deliver personalized respiratory support and potentially improve patient outcomes.

## 1. Introduction

Acute respiratory distress syndrome (ARDS) is a life-threatening form of acute hypoxemic respiratory failure (AHRF), and it represents a prominent cause of admission to intensive care units (ICUs) [1]. The hallmarks of ARDS are impaired gas exchange (mainly hypoxemia) [2,3] and lung injury, which are characterized by diffuse inflammatory infiltrates, alveolar flooding, and atelectasis with decreased lung compliance [4]. The definition of ARDS has been slightly changed since its first description in 1967 [5], and the most recent classification dates back to the “Berlin definition” in 2012 [6]. This definition focused on the following: recent onset (within one week of a known insult); hypoxemia, which is assessed at a minimum positive end-expiratory pressure (PEEP) of 5 cmH_2_O via the O_2_ partial arterial pressure (P_a_O_2_) to the inspired oxygen fraction (F_I_O_2_) ratio (graded as mild (P_a_O_2_/F_I_O_2_ < 300), moderate (P_a_O_2_/F_I_O_2_ < 200), or severe (P_a_O_2_/F_I_O_2_ < 100)); bilateral pulmonary infiltrates in the chest, which are assessed via X-ray; and non-cardiogenic and non-fluid overload-related pulmonary edema. From an epidemiologic standpoint, the LUNG SAFE study [1] revealed that ARDS affects 23% of critically ill patients and requires mechanical ventilation (10% of ICU patients); moreover, it burdened by a hospital mortality of 46% for the most severe cases. However, recent data [7] shows that more than 93% of patients with AHRF receive high-flow oxygen therapy (HFOT, which is a noninvasive respiratory support that is not included among the diagnostic criteria of ARDS) and then continue on to have a P_a_O_2_/F_I_O_2_ < 300 after intubation and when under positive pressure invasive mechanical ventilation (IMV), thus implying a greater incidence of ARDS than currently reported [1]. In this setting, the mainstay for the management of ARDS consists of noninvasive [8] and invasive [9] respiratory support strategies, whose specific and reciprocal roles are unclear but evolving in accordance with the understanding of biological [10], radiological, and clinical heterogeneity (e.g., insult events, secondary organ dysfunctions, and the severities of illness) [11,12,13] of the disease.

In this narrative review, we will provide an overview of noninvasive and invasive respiratory support strategies in the context of ARDS. Specifically, we will focus on ARDS sub-phenotypes that have specific peculiarities of respiratory system mechanics, and this may orient a personalized approach to respiratory support strategies.

## 2. The Concept of Baby Lung

The concept of “baby lung” was introduced by Gattinoni and Pesenti in 1987 [14], and it represents a model through which to explain the mechanical characteristics of injured lungs in the setting of ARDS. By the systematic use of CT-scans, the authors demonstrated that the amount of normally aerated lung tissue at end expiration was mainly located in the nondependent lung region: its size correlates with respiratory compliance and is dimensionally equivalent to the pulmonary size of a healthy young boy. Conversely, the number of nonaerated, consolidated, and collapsed lung tissue was mainly located in the dependent lung region, and it was correlated with hypoxemia, shunt fraction, and pulmonary hypertension. In this context, respiratory compliance represented a reliable estimation of the baby lung size, whose mechanical characteristics were nearly normal [15,16,17]. This anatomical model was challenged by the observation that the prone position led to a redistribution of the baby lung from non-dependent to dependent regions [18,19]. The pathophysiology of this phenomenon relies on the widespread distribution of inflammatory edema, and this implies an increased lung weight exerting a hydrostatic pressure (superimposed pressure) according to a vertical gravitational gradient [4,20]. Accordingly, aerated lung regions are squeezed and compressed by the heavy parenchyma above, thus justifying the redistribution of the baby lung after a body position change [4]. These findings led researches to recognize the concept of baby lung as a functional model [4,20], where the lung basically acts as a “sponge” [21].

### 2.1. Patient Self-Inflicted Lung Injury (P-SILI)

**Figure 1:**
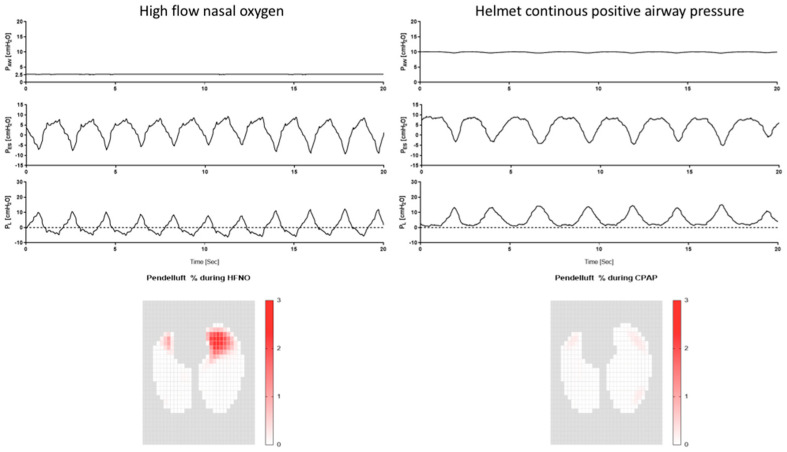
Traces of airway pressure, esophageal pressure, and transpulmonary pressure for a patient undergoing treatment with high-flow nasal oxygen (**left panel**) and helmet continuous positive airway pressure (**right panel**). In the (**bottom panels**), the pendelluft effect is depicted: areas with a bright red color indicate a high pendelluft effect, while the white regions represent no pendelluft. The percentage scale relates to the total tidal volume. Despite the patient exerting a similar inspiratory effort, resulting in the same transpulmonary pressure during both the high flow and the helmet phase, the PEEP administered via the helmet effectively decreased the pendelluft effect, as shown in the (**bottom panels**).

Patient self-inflicted lung injury (P-SILI) refers to the harmful effect of spontaneous breathing on injured lungs [22,23,24], and it is characterized by increased respiratory drive and intense inspiratory effort (with or without tachypnea). These phenomena, coupled with the regional heterogeneity of lung compliance, cause vigorous transpulmonary pressure swing (ΔP_L_) with regional overdistention, excessive transpulmonary pressure, and atelectrauma [25,26,27]. Specifically, an increased inspiratory drive generates larger tidal volume (V_T_), leading to baby lung hyperinflation, consequent mechanical distortions, and injury [28]. Moreover, the inhomogeneous distribution of ΔP_L_ generates alveolar pressure gradients and gas displacement from the ventral non-dependent to the dorsal-dependent lung regions (i.e., pendelluft) [29], and this occurs with a consequent hyperinflation and damage of the latter (Figure 1). Finally, the intense inspiratory effort exerted on the interstitial space increases the transmural vascular pressure and leads to hydrostatic pulmonary edema [24,30].

### 2.2. Ventilator-Induced Lung Injury (VILI)

The pathophysiology of VILI relies on the inappropriate management of IMV neglecting the evolving characteristics of lung mechanics (size and elastic properties), thus causing volutrauma, barotrauma, and atelectrauma [31]. Specifically, a mechanical ventilator impresses a certain amount of force (as volume and pressure) to the skeletal structure of the lung, which is composed of extensible elastin and inextensible collagen, thus leading to progressive fiber elongation (strain) and tension (stress). Volutrauma is caused by a non-physiological strain due to hyperinflation, and it induces mechanoceptor activation, cytokine production, and worsening inflammation [4]. Barotrauma develops when stress overwhelms the tensile properties of the pulmonary fibers that lead to parenchymal rupture (e.g., pneumothorax [32]). Atelectrauma is caused by the repetitive opening and closing of airways and lung units [33]. For these reasons, a physiology-based, patient-centered, personalized approach to IMV has been strongly advocated for [6,9].

## 3. Noninvasive Support

### 3.1. The Benefits and Harms of Maintaining Spontaneous Breathing

Noninvasive respiratory support (NIRS) includes standard oxygen therapy (SOT), HFOT, continuous positive airway pressure (CPAP), and noninvasive ventilation (NIV) [34]. These strategies share in being externally applied to the upper airways, thus preserving the physiological mechanisms of respiratory system protection (e.g., gas clearance, cough, secretion mobilization, and drainage) [35,36] and spontaneous breathing, with improved ventilation of the dorsal-dependent lung regions [37]. Moreover, the application of NIRS may prevent clinical complications that are associated with intubation and IMV, such as ventilator-associated pneumonia (VAP) [36], delirium [38], and muscular weakness [39]. However, intubation and IMV are required in 30–60% of patients that are initially treated with NIRS. Most importantly, NIRS failure is independently associated to increased mortality [40,41,42], which may be reasonably explained by the development of P-SILI during the treatment. 

### 3.2. Strategies and Setting

#### 3.2.1. High-Flow Oxygen Therapy

HFOT delivers a heated and humidified gas flow mixture of oxygen and air up to 60 L/min, with a set F_I_O_2_ up to 100% through the large bore nasal cannula (i.e., high-flow nasal oxygen therapy, HFNOT) [34,43,44] (Table 1). The main physiological effects of HFOT are as follows: the accurate delivery of a set F_I_O_2_ that matches the patient’s peak inspiratory flow and allows for a reliable evaluation of the P_a_O_2_/F_I_O_2_ ratio [7]; a washout of nasopharyngeal dead space, which increases ventilatory efficiency and reduces the work of breathing; a flow-dependent positive pressure effect (3–5 cmH2O), which allows for lung recruitment, improved oxygenation, and improved lung mechanics; the active humidification and heating of the upper airways, favoring secretion hydration and clearance; and patient’s comfort [45,46].

#### 3.2.2. Continuous Positive Airway Pressure

CPAP provides a constant positive pressure to the upper airways via face-mask or helmet interfaces [47] (its detailed description is summarized in Table 1 [34,43]). Briefly, the positive pressures of 5–8 cmH_2_O for face masks (higher pressures may lead to proportionally increased air leakage with consequent mismatches between set and delivered pressures, which potentially implies a lower efficacy for this therapy) and 10–15 cmH_2_O for helmets are generated by a flow generator (compressed gases or turbine) or a Venturi system that provides a continuous fresh gas flow to the inlet port while the outlet port is regulated by a PEEP valve [34,43]. In this setting, a minimum gas flow of 40–60 L/min (>35 L/min) is necessary to reduce the risk of CO_2_ rebreathing [48]. Accordingly, the ventilator-derived CPAP should be avoided (<30 L/min), especially with the helmet interface [47,48,49]. Whenever possible, setting an appropriate flow-by on the machine is recommended to overcome this issue. Moreover, active humidification is necessary to maintain an adequate level of air humidity (15 mgH_2_O/L). 

The CPAP exerts beneficial physiological effects via the improvement of functional residual capacity (FRC) and through the reduction in airway resistance (by increased airway pressure, thus preventing airway collapse and flow limitation [34]), which leads to improved oxygenation [50,51,52,53,54,55].

#### 3.2.3. Noninvasive Ventilation

NIV allows for the application of a biphasic positive airway pressure (PEEP + pressure support, PS). It is generated by a mechanical ventilator and delivered via face-mask or helmet interfaces [34,43,44] (Table 1). Active humidification is recommended only for face-mask NIV, while it is not required for helmet-NIV when the total system minute ventilation exceeds 40 L/min [28,56,57]. During NIV, the physiological benefits exerted by CPAP are implemented by the PS (8–14 cmH_2_O)—which unloads respiratory muscles, thus decreasing inspiratory effort and the work of breathing [51,58]. However, full respiratory synchronization may increase P_L_, ΔP_L_, and V_T_ [59,60], thus increasing the risk of P-SILI. In comparison with facemask-NIV, this phenomenon may be attenuated during helmet-NIV because a proportion of the pressure is dissipated when distending the interface. This condition causes a trigger delay (0.1–0.5 s) that leads to inspiratory desynchronization, which is sub-optimal for muscle unloading but exerts lung-protective effects limiting the amplitude of P_L_ swings [61], thus possibly reducing the risk of P-SILI. Moreover, helmet-NIV is associated with expiratory desynchronization, which causes a patient’s expiration against the expiratory pressure above the set PEEP, and this might contribute to alveolar recruitment [61].

### 3.3. Clinical Evidence

All these strategies are effective for improving hypoxemia, and no significant differences were demonstrated among the patients with mild ARDS (P_a_O_2_/F_I_O_2_ > 200) [62]. In contrast, current guidelines [8] recommend the use of HFOT as the first-line intervention for patients with moderate-to-severe ARDS. Recently, Ospina and colleagues [63] compared the effects of SOT vs. HFOT in patients with severe COVID-19 ARDS. They found that HFOT significantly reduced the intubation rate and shortened the median time for clinical recovery. However, growing evidence has suggested a role for CPAP and Helmet-NIV for the management of hypoxemic patients with moderate-to-severe hypoxemia [64]. Specifically, the RECOVERY-RS clinical trial [65] enrolled 1273 patients with COVID-19 ARDS from 48 hospitals, who were randomized to receive CPAP, HFOT, or SOT treatments. This study found that CPAP (delivered with various interfaces at the discretion of attending physicians) significantly reduced the composite outcome of 30-day mortality and intubation rate compared with SOT [65]. On top of this, a network metanalysis [64] of 25 randomized clinical trials found that helmet-NIV was associated with a lower risk of intubation compared with SOT (RR, 0.26 [95% CI, 0.14–0.46]), HFOT (RR, 0.35 [95% CI, 0.18–0.66]), and facemask-NIV (RR,0.35 [95% CI,0.19–0.61]). These results were partially confirmed by Grieco et al. [66], who randomly assigned critically ill patients with moderate-to-severe COVID-19 ARDS to helmet-NIV vs. HFOT, and found that there were no different number of days that were free of respiratory support (although in patients receiving the helmet-NIV, the intubation rate was lower (30% vs. 51%, respectively, *p* = 0.03)). In contrast, Arabi et al. [67] randomized 320 patients with COVID-19 AHRF to helmet-NIV vs. the usual composite respiratory support (a combination of facemask-NIV, HFOT, and SOT) and found no different mortality rates between the study groups. However, the imprecise effect estimate and the lack of direct comparison between these strategies limited the reproducibility and the external validity of results. A multicenter, randomized controlled trial is currently ongoing and will shed light of the effect of HFOT, CPAP, and NIV on the intubation rate of critically ill patients with moderate-to-severe AHRF (NCT05089695).

### 3.4. Physiological Rationale for Using High PEEP

The benefits associated with helmet-CPAP and helmet-NIV in patients with AHRF and ARDS are mostly related to the alveolar recruitment that is induced by high PEEP (≥10 cmH_2_O) when it is applied for a longer period of time (≥48 h), which increases the FRC and allows for the homogeneous distribution of V_T_, thus improving the P_L_, the ΔP_L_, and the ventilation-to-perfusion ratio [68]. Moreover, the PEEP-induced FRC increasingly mitigates atelectrauma [27], reduces the occurrence of pendelluft [22,30,69], and flattens the diaphragm [70], thus promoting neuromechanical uncoupling with a consequent mitigation of the dynamic strain for a given inspiratory drive. Additionally, diaphragm flattening may prevent the diaphragm dysfunction that is caused by the excessive concentric contractions that are due high inspiratory effort [71], although this does warrant additional clarification through further investigation. Furthermore, the PS-induced reduction found in the work of breathing lowers the oxygen consumption with a consequent improvement of hypoxemia [72,73]. 

### 3.5. How to Assess the Safety of Spontaneous Breathing

**Figure 2:**
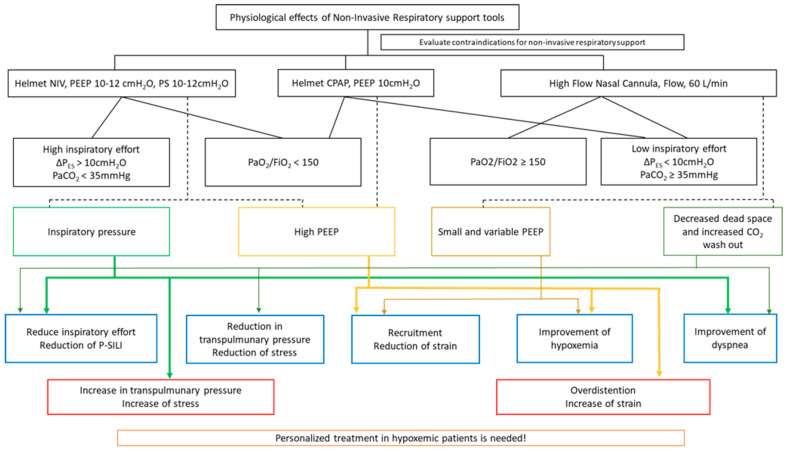
Flow chart of the physiological effects of helmet-CPAP, helmet-NIV, and HFNO. This figure shows the most common settings and the main physiological effects of each noninvasive respiratory support.

The current definition of ARDS is based on the clinical impact of lung injury on gas exchange, whose characteristics evolve over time and which require specific supportive interventions that are aimed at mitigating the progression of P-SILI. For this reason, NIRS should be oriented by a personalized approach that is titrated on the intensity of the respiratory effort assessed by both pleural pressure swing (ΔP_PL_), which is estimated by the esophageal pressure swing (ΔP_ES_) [74], and surrogate measurements (e.g., P_a_CO_2_ < 35 mmHg) [75]. Specifically, Grieco et al. [61] conducted a randomized cross-over study to investigate the physiological effect of HFOT vs. helmet-NIV in critically ill patients with moderate-to-severe AHRF. In comparison with HFOT, helmet-NIV significantly reduced ΔP_ES_, although it led to a ΔP_L_ increase in patients who exhibited a lower inspiratory effort (ΔP_ES_ < 10 cmH_2_O) at baseline. In accordance with these findings, a post hoc analysis of the HENIVOT trial [76] confirmed the beneficial effect of helmet-NIV over HFOT in patients who exhibited a high respiratory drive (which was assessed by hypocapnia (P_a_CO_2_ <35 mmHg)) and P_a_O_2_/F_I_O_2_ to a numerical dyspnea rating scale of <30 at baseline. A recent cross-over randomized trial compared the physiological effects of helmet-CPAP vs. helmet-NIV vs. HFOT in critically ill patients with AHRF [77]: in comparison with HFOT, helmet-CPAP and helmet-NIV led to oxygenation improvement, increased V_T_, and end-expiratory lung volume. However, helmet-NIV decreased the ΔP_ES_ in those who underwent intense inspiratory effort (ΔP_ES_ > 10 cmH_2_O) before treatment start (possibly due to the reduction in the respiratory muscle workload), but there was increased P_L_ and ΔP_L_ in those with low inspiratory effort (ΔP_ES_ < 10 cmH_2_O) (which possibly favored the progression of P-SILI). Interestingly, the authors showed that the ventilatory heterogeneity caused by pendelluft are frequent during spontaneous breath, and can be mitigated by the application of high PEEP through the helmet interface. Taken together, these findings imply that HFOT and CPAP are effective for improving oxygenation in patients with a less severe form of AHRF, which is characterized by low inspiratory effort (ΔP_ES_ < 10 cmH_2_O) and/or normocapnia. CPAP improves alveolar recruitment and limits the occurrence of pendelluft. In contrast, helmet-NIV may play a role in the management of patients with severe AHRF, who are characterized by intense inspiratory effort (ΔP_ES_ > 10 cmH_2_O) and/or hypocapnia (P_a_CO_2_ < 35 mmHg). Accordingly, the physiologic characterization of respiratory mechanics (Figure 2) is pivotal for identifying patients who benefit the most from each strategy, and this is required in order to personalize supportive management and to potentially optimize the clinical outcome.

### 3.6. Monitoring Tools of NIRS Failure

**Figure 3:**
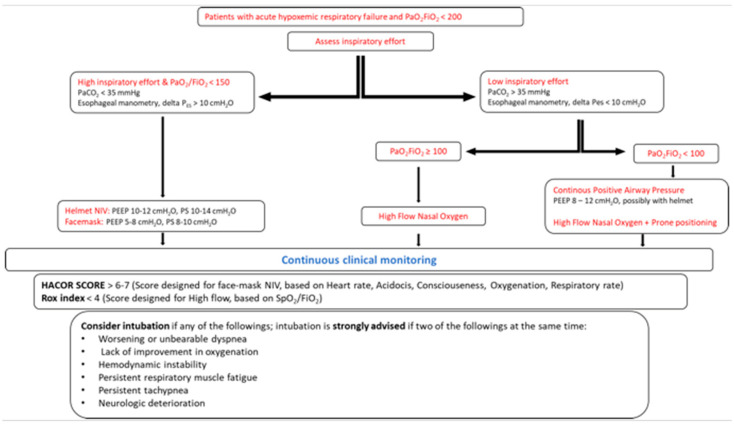
This flowchart illustrates a proposed treatment protocol for acute hypoxemic respiratory failure, and is based on the patient’s clinical presentation and phenotypes (from the authors’ perspective). The first step entails assessing the inspiratory effort, ideally through direct measurements (via an esophageal balloon) or alternatively through indirect measurements (e.g., PaCO2 < 35 mmHg in the absence of metabolic acidosis).

Patients with ARDS and AHRF warrant close monitoring to sooner detect the signs of disease progression and lack of benefit from the delivery of NIRS. This is required in order to prevent the potential harm caused by delaying intubation and IMV [78]. In this setting, an increasing amount of evidence has shown that the delayed recognition of NIRS failure is associated with increased mortality, and this is possibly due to the progression of P-SILI [8]. Accordingly, specific clinical tools have been implemented in daily practice to overcome this issue and were demonstrated to be effective for the early recognition of NIRS failure (Table 2, Figure 3). 

Respiratory rate and blood oxygenation are monitored by both continuous pulse oximetry and intermittent arterial blood gas analysis, and they may play an important role for the purpose of identifying patients at risk of NIRS failure [25,26,79]. A progressive increase in respiratory rate and/or a worsening of oxygenation over time correlates with lung function deterioration, and it demands intubation and IMV to prevent the progression of P-SILI. Although these parameters represent the standard of care for an initial monitoring of AHRF response to NIRS at the bedside, their low sensitivity and specificity for identifying treatment failure imply the application of advanced tools to titrate the clinical management to patient needs:

- An expired tidal volume of >9–9.5 mL/kg predicts the body weight and may predict facemask-NIV failure [80], but it is not applicable during helmet-NIV;
- The inspiratory effort assessed by ΔP_ES_ > 15 cmH_2_O or lack of early ΔP_ES_ reduction over time (within the first 2 h of treatment) may predict NIV failure at 24 h [81];
- The ROX index, defined as the ratio of SpO_2_/F_I_O_2_ to respiratory rate, may identify patients at risk of HFOT failure and the need for intubation and invasive mechanical ventilation [82]. Additionally, the ROX index has been recently demonstrated to moderately predict NIV failure in patients with AHRF [83];
- The HACOR scale takes into account heart rate, acidosis, stream of consciousness, oxygenation, and respiratory rate, with highest possible score of 25 points. Specifically, a score of 5 as the cutoff has a good diagnostic accuracy for identifying patients at risk of NIV failure in the different subgroups classified for diagnosis, age, disease severity, and those at a different timepoint. In those patients with a HACOR score > 5 at 1 h after NIV initiation, early intubation (≤12 h) may decrease hospital mortality [79]. Recently, Duan et al. [84] reported a significantly improved predictive power for NIV failure in an updated version of the HACOR scale that takes into account six baseline variables (pneumonia, cardiogenic pulmonary edema, pulmonary ARDS, immunosuppression, septic shock, and the SOFA score). Patients with updated HACOR scores of ≤7, 7.5–10.5, 11–14, and >14 were classified at a low, moderate, high, and very high probability of NIV failure.

In scenarios that are characterized by high inspiratory effort, noninvasive ventilation (NIV) could offer physiological benefits such as reduced inspiratory effort (potentially minimizing P-SILI) and enhanced lung homogeneity (due to the applied PEEP).

On the other hand, for cases of low inspiratory effort, the chosen intervention depends on the severity of hypoxemia. If the patient exhibits profound hypoxemia, there may be potential benefits from the high PEEP provided by helmet-CPAP or from the prone positioning during spontaneous breathing; otherwise, the use of high-flow nasal oxygen is advised.

## 4. Invasive Mechanical Ventilation

### 4.1. Main Aims

For patients with AHRF and ARDS failing NIRS, the IMV is pivotal for allowing physiologic gas exchange, as well as lung and diaphragm protection whenever the respiratory demand (e.g., respiratory drive) overwhelms the respiratory system capacity (e.g., inspiratory efforts). Accordingly, IMV is a supportive intervention that allows time for lung recovery while preventing the progression of P-SILI [23]. Although IMV, since its first large-scale use during the polio epidemic in 1952 [85], has been used to provide adequate blood oxygenation (P_a_O_2_ > 60 mmHg or SpO_2_ within 90–94%) and maintaining the appropriate P_a_CO_2_ levels for targeting acid–base balance homeostasis (pH within 7.35–7.45), several studies have shown that it may harm patients by leading to VILI [5,32]. 

### 4.2. Controlled Mechanical Ventilation

Controlled IMV is the cornerstone for the management of severe ARDS [6], which implies respiratory muscle paralysis [86] or apneic ventilation [87]. It is delivered in order to mitigate excessive pleural pressure (P_PL_) swings and heterogeneity, and these result from the interplay between inspiratory efforts (P_mus_) and reduced alveolar units with normal compliance [4]. According to the equation of motion that includes elastic, as well as resistive and static pressure components, the airway pressure (P_AW_) results from the sum of the P_L_ and P_PL_, whose main determinant is the elastic recoil pressure of chest wall (P_CW_).

P_AW_ = P_L_ + P_PL_

In a seminal experimental model, Dreyfuss et al. [88] demonstrated that VILI was not determined by high P_AW_ per se but developed from the detrimental effect of lung overdistention and the P_L_ increase caused by a large V_T_. This highlights the importance of transpulmonary rather than absolute airway pressure in determining VILI.

### 4.3. How to Set VT

**Figure 4:**
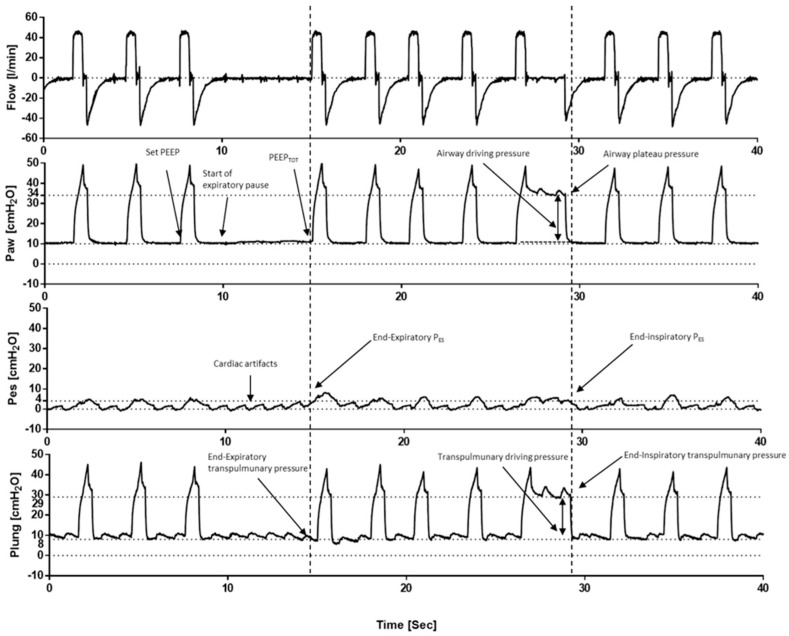
Respiratory system mechanics during volume-controlled ventilation. The end-expiratory hold allows for an assessment of the total positive end-expiratory positive pressure (PEEP), which results from the sum of the PEEP set on mechanical ventilators and the intrinsic PEEP (PEEPi). Contemporary esophageal manometry shows the end-expiratory esophageal pressure (PES), thus allowing for the measurement of transpulmonary pressure (P_L_) via the equation of motion. The end-inspiratory hold allows for the assessment of the plateau pressure (P_PLAT_) and the driving pressure (ΔP).

Assuming that all alveoli are opened, and that the contribution of the chest wall is negligible (e.g., non-obese patients), the P_L_ may be estimated by the plateau pressure (P_PLAT_) (Figure 4), which is the P_AW_ displayed by the ventilator after an inspiratory hold of 0.3 sec. In critically ill patients with ARDS, a low V_T_ (6 mL/predicted body weight, PBW) ventilation with low P_PLAT_ (<30 cmH_2_O) was demonstrated to be effective for reducing mortality compared with a high VT (12 mL/predicted body weight) ventilation with high P_PLAT_ [89,90]. Titrating V_T_ to reach an upper P_PLAT_ limit within 28 cmH_2_O was demonstrated to be effective for further reducing the risk of overdistention [91]. Subsequent data have indicated that normalizing V_T_ to the respiratory system compliance rather than predicted body weight allows one to assess the mechanical distortion of the baby lung induced by V_T_. This parameter is the driving pressure (ΔP = V_T_/C_RS_) and can be easily measured at the bedside as P_PLAT_-PEEP (Figure 4) [92]. Specifically, Amato et al. [93] reported an independent association between a ΔP < 15 cmH_2_O and improved survival, and this was not influenced by PEEP and V_T_ (Table 3). Moreover, Gattinoni et al. [94] introduced the concept of mechanical power, which is the energy transmitted to the respiratory system by the mechanical ventilator with the aim of aggregating the effect of the ventilatory variables contributing to VILI into a single measure. However, this interesting model raised several concerns [95,96,97] due to the fact that PEEP is a static pressure, the “weight” of each parameter on the development of VILI and death was not balanced, and the different effect of PEEP among recruiters (PEEP-induced FRC increase) and non-recruiters was not characterized. Moreover, the mechanical power calculation lacks feasibility at the bedside and does not orient the clinician to best manage IMV in order to prevent its burden. In contrast, Costa et al. [96] weighted the effect of each component by determining the mechanical power on mortality in patients with ARDS. The authors found that mechanical power, ΔP, and respiratory rate (RR) were significant predictors of mortality, and a simpler model (4 × ΔP + RR) was equivalent to mechanical power predicting mortality. However, the clinical benefit of this physiologic hypothesis warrants demonstration in future randomized clinical trials.

### 4.4. How to Set PEEP

Low V_T_ ventilation may induce alveolar de-recruitment, which leads to oxygenation impairment and can be theoretically reversed by PEEP [98,99]. However, ARDS is a complex clinical syndrome characterized by several degrees of lung inhomogeneity, whose morphological (diffuse vs. focal infiltrates) and biochemical (hyperinflammatory vs. immunosuppressive) characteristics may identify the specific sub-phenotypes with different responses to PEEP [13]. Specifically, patients with diffuse ARDS and hyperinflammation were more likely to benefit from higher PEEP levels [100], thus reducing both the dynamic strain and the risk of atelectrauma for a given V_T_ [101]. Conversely, patients with focal ARDS and a non-hyperinflammatory phenotype were more likely to benefit from lower PEEP levels, which mitigate the dynamic strain caused by the regional overdistension and hemodynamic impairment that are due to preload dependency and right ventricular failure [102,103]. For these reasons, several pragmatic clinical trials that randomized patients with ARDS to receive lower vs. higher PEEP based on oxygenation- [104,105] and C_RS_-oriented [106,107] criteria failed to identify the best strategy to set and individualize PEEP in daily practice. Nonetheless, a systematic review and meta-analysis [108] on 2299 patients from three clinical trials found that the application of higher PEEP levels in the most severe patients with ARDS was associated with an improved survival, while a randomized controlled trial [109] demonstrated that a ventilator strategy misaligned to lung morphology may increase mortality. Currently, the gold standard method for assessing lung morphology and recruitability is represented by the CT-scan (Table 3), whereas its use in daily practice appears poor in terms of feasibility due to personnel shortage, increased workload, and health system costs. For these reasons, new tools for PEEP titration at the bedside have been implemented in clinical practice (Table 3), although their role must be clarified in future clinical trials:

- The recruitment-to-inflation ratio (R/I): this index reflects the amount of recruited lung units that are normalized to the C_RS_ during a single-breath de-recruitment maneuver [110] from high PEEP (15 cmH_2_O) to low PEEP (5 cmH_2_O) while taking into account the airway opening pressure [111]. Briefly, a R/I ratio above 0.5 identifies patients for whom a higher PEEP level increases the FRC with negligible alveolar hyperinflation, while a R/I ratio below 0.5 identifies those who develop PEEP-induced hyperinflation and may benefit from lower PEEP levels. A clinical trial investigating whether a PEEP-setting strategy based on the R/I ratio can improve clinical outcome in ARDS is currently ongoing (NCT03963622).
- Electrical impedance tomography (EIT): this is a noninvasive, radiation-free imaging method that tracks the global and regional lung volume changes induced by PEEP. EIT shows good reliability in the assessment of lung recruitment vs. hyperinflation compared with CT-scans [112]. For instance, in a supine position, the percent of the dorsal-to-ventral thorax diameter is expressed as the center of ventilation (COV) [113], and this may help to describe the distribution of VT between the ventral nondependent aerated lung regions (COV > 50%) vs. dorsal-dependent non-aerated lung regions (COV < 50%). Accordingly, a COV > 50% may be a marker of the inhomogeneous VT distribution that is associated with a high risk of ventral hyperinflation and dorsal atelectasis. Moreover, EIT provides functional information on the recruitable alveolar collapse by measuring changes in pixel compliance via a decremental PEEP trial: a decreased pixel compliance when lowering PEEP is suggestive of collapse, thus indicating potential for recruitment, whereas increased pixel compliance is suggestive of overdistention [112]. Future clinical investigations in this context are urgently needed.
- Esophageal manometry: this method measures Pes, which is an estimation of PPL in the mid-thorax region adjacent to the esophageal balloon. A recent validation study on supine pigs and human cadavers [114] showed that injured lungs exhibit a vertical P_PL_ gradient, which increases from ventral non-dependent regions to dorsal-dependent lung regions. The P_L_ can be estimated by the equation of motion that substitutes P_PL_ with the end-expiratory P_ES_ and by the elastance-derived method at end inspiration, whose value is representative of the non-dependent part of the chest cavity [74,102]. In this context, a post hoc analysis of the EPVent-2 (esophageal pressure-guided ventilation 2) trial [115] found a significant improvement in ventilator-free, shock-free days and in the survival rates, regardless of the treatment group, among patients receiving PEEP and yielding a positive end-expiratory P_L_ close to 0 cmH_2_O. This finding may be particularly of interest in the management of obese patients with considerable chest wall elastance that leads to high ΔP, even when the P_L_ remains within safe limits [116]. In this context, a PEEP-setting strategy for obtaining a positive end-expiratory PL was associated with survival improvement in a large multicenter study [117].
- Volumetric capnography (Vcap) is a noninvasive tool that may help to assess the amount of alveolar and airway dead space [118], which are directly associated with increased mortality in patients with ARDS [119]. In ARDS patients, preliminary evidence has suggested a role for Vcap in PEEP titration in terms of reaching the highest compliance in conjunction with the lowest ratio of dead space to V_T_ [120]. Although this tool may provide important insight into lung mechanics, especially when esophageal manometry is not available at the bedside, it warrants further investigation to clarify its role in the setting of ARDS.

### 4.5. How to Assess the Safety of Assisted Invasive Mechanical Ventilation

**Figure 5:**
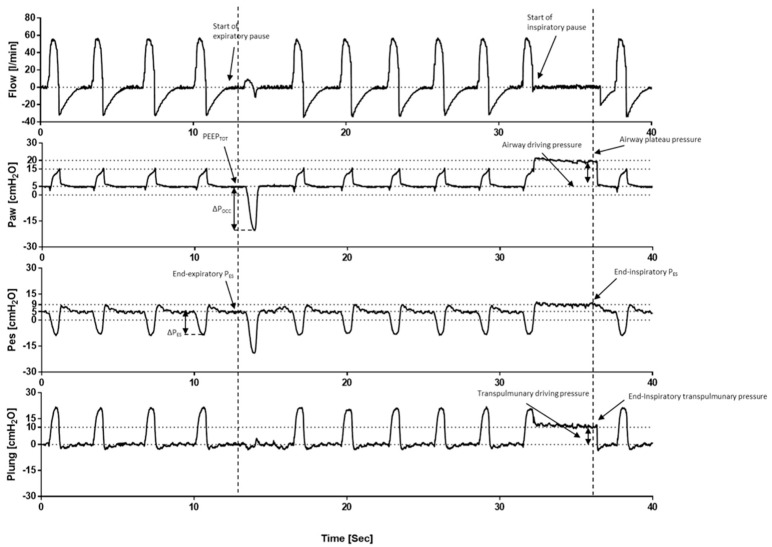
Respiratory system mechanics during assisted ventilation.

The transition from controlled to assisted mechanical ventilation should be promoted as soon as it appears safe in order to prevent respiratory muscle dysfunction and atrophy, as well as the further complications associated with prolonged sedation (e.g., delirium, stress ulcers, pneumonia). Nonetheless, patient–ventilator interactions should be monitored carefully (Figure 5) to sooner detect asynchronies [121], mitigate over-assistance, and to improve the under-assistance that leads to excessive P_L_ and consequent lung injury. For these reasons, ventilator assistance should be titrated to respiratory drive, inspiratory effort, and lung mechanics [122]. Specifically, low-respiratory drive and effort may be a consequence of over-assistance and/or excessive sedation, or diaphragm dysfunction [74]. In this context, propofol and benzodiazepines were demonstrated to be effective in reducing respiratory effort [123,124], while opioids lower the respiratory rate with mixed effects on the effort [125,126], and dexmedetomidine plays no role in the management of respiratory drive [127]. The gold standard parameter for assessing respiratory effort is represented by the negative deflection of P_ES_, whose magnitude estimates the strength of effort. Furthermore, its integral over inspiratory time quantifies the energy expenditure (PTP_ES_) and its swing measures the driving P_L_, thus stratifying the risk of P-SILI [128]. However, esophageal manometry requires specific equipment and expertise that may limit its use in daily clinical practice. In this context, occlusion maneuvers (Figure 5) may play a role, as any P_AW_ changes follow the magnitude and timing of P_PL_ variations, and they are independent from respiratory mechanics when air flow is equal to 0 (Table 3). The P_AW_ drop during the first 100 msec of the occluded breath (P_0_._1_) is a measurement of respiratory drive [129], and it normally ranges between 1–4 cmH_2_O, while higher values may be considered as surrogates of under-assistance or dysregulated respiratory drive. In contrast, a low respiratory rate may be a sign of over-assistance. Furthermore, the P_AW_ drop during a whole occluded breath (P_OCC_) is a measurement of inspiratory effort, and its normal value ranges between 10 and 15 cmH_2_O [130]. Moreover, the assessment of P_PLAT_ (Table 3) was demonstrated to be effective in stratifying the risk of hyperdistention and was directly associated with mortality [131]. 

The end-expiratory hold allows for the measurement of P_0_._1_ within the first 100 msec and for the airway pressure variation within the whole occluded breath (ΔP_OCC_). These respectively measure the inspiratory drive and effort. The end-inspiratory hold allows for the measurement of the airway plateau pressure and the esophageal plateau pressure. By calculating the transpulmonary plateau pressure and the transpulmonary driving pressure, we can gain insight into the patient’s lung function and the effectiveness of the pressure support ventilation. It should be noted that during the end-inspiratory hold there is an increase in esophageal pressure above the end-expiratory pressure, which is an effect of the chest wall elastance (and this allows one to calculate chest wall mechanics).

## 5. Neuromuscular Blockage, Prone Position, and Inhaled Pulmonary Vasodilators

Besides a physiology-based NIRS delivery and mechanical ventilator settings, other interventions have been demonstrated to be effective in improving the outcome of ARDS patients. 

### 5.1. Neuromuscular Blocking Agent

Neuromuscular blocking agent (NMBA) administration (specifically, Cisatracurium) for 48 h was demonstrated to be effective for reducing 90-day mortality in patients with early moderate-to-severe ARDS compared with placebo [86]. In contrast, the ROSE trial [87] showed no differing 90-day mortality rates between patients who were randomized to receive either deep sedation with Cisatracurium for 48 h or lighter sedation without NMBA infusion (intermittent NMBA boluses were allowed as for clinical indication). In light of these studies, a recent clinical practice guideline [39] was suggested to avoid a continuous NMBA infusion for patients with ARDS of any severity and who are being ventilated with a lighter sedation strategy. However, for those who need deep sedation to control inspiratory effort, a short-term (48 h) infusion of these drugs represents a reasonable option for facilitating lung protective ventilation.

### 5.2. Prone Position

Prone positioning sessions of at least 16 h were demonstrated to be effective for reducing 28-day mortality in mechanically ventilated patients with moderate-to-severe ARDS compared with placebo [132]. Furthermore, these results were confirmed by a subsequent meta-analysis on eight randomized controlled trials (2129 patients) [133]. A physiological explanation relies on the prone-positioning-induced recruitment of dependent lung regions, and this implies the following: a mitigation of ventilation to perfusion mismatches, which leads to improved oxygenation; a more homogeneous distribution of tidal volume; and improved lung compliance and reduced P_L_ with mechanical ventilator-induced stress and strain modulation [134].

### 5.3. Inhaled Pulmonary Vasodilators

Nitric oxide has been widely used in clinical practice to improve ventilation to perfusion mismatches and for reducing pulmonary hypertension [135]. A recent systematic review and meta-analysis [136] found that nitric oxide administration compared with controls did not increase overall survival (13 randomized controlled trials, 1243 patients), 28-day survival (9 randomized controlled trials, 1105 patients), nor bleeding events (5 randomized controlled trials, 614 patients). Although nitric oxide administration improved oxygenation (11 randomized controlled trials, 614 patients), it increased the risk of renal impairment in adults (4 randomized controlled trials, 9455 patients). For these reasons, the evidence is insufficient for supporting nitric oxide administration in this setting.

## 6. Potential Issues for the Implementation of Personalized Respiratory Support Strategies and Eventual Solutions

The LUNG SAFE [1] trial showed that ARDS is underdiagnosed (34% of the cases at the time of fulfilment of ARDS criteria), and its recognition is frequently delayed. Factors associated with the clinician recognition of ARDS were higher in the nurse-to-patient ratio, younger patient age, lower P_a_O_2_/F_I_O_2_ ratio, and pneumonia or pancreatitis variables, while the absence of risk factors for ARDS and concomitant cardiac failure predicted a reduced likelihood of clinician recognition. Moreover, this study reported that 35.1% of patients with ARDS did not receive protective mechanical ventilation, and only 16.3% of those with severe ARDS underwent prone positioning, and the P_PLAT_ was poorly measured (40.1% of patients). Accordingly, a first step toward an implementation of personalized respiratory support strategies relies on making clinicians aware of ARDS diagnostic criteria in order to allow an early diagnosis and to allow for a prompt delivery of appropriate respiratory strategies. Moreover, it is worthwhile to educate clinicians in the use of the bedside clinical tools discussed above, whose application may help to sooner recognize NIRS failure and to accurately titrate the mechanical ventilator setting to lung mechanics. 

## 7. Conclusions

ARDS and AHRF are heterogeneous clinical conditions characterized by peculiar features of respiratory system mechanics. The management of invasive and noninvasive respiratory support strategies in terms of neglecting the respiratory system physiology may perpetuate the progression of lung injury. A personalized and physiology-based approach to ARDS and AHRF is strongly advocated to limit the evolution of lung injury and to provide enough time to recover. Future trials are justified to verify this hypothesis and to test whether such an approach may improve the outcome of these life-threatening clinical conditions.

## Figures and Tables

**Table 1:** The main settings, benefits, and pitfalls of noninvasive respiratory supports in patients with AHRF/ARDS.

Noninvasive Respiratory Supports		Settings	Benefits	Pitfalls
High-flow oxygen therapy		- F_I_O_2_: 0.21–1 - Gas flow: 40–100 L/min - Gas temperature: 31–37 °C	- Patient’s inspiratory flow matches - The reliable delivery of conditioned gas at the F_I_O_2_ set by the clinician - Positive airway pressure (up to 4 cmH_2_O) - Washout of nasopharyngeal dead space - Relieved inspiratory effort - Enhanced patient comfort	- Delivery of small PEEP levels
CPAP/NIV	Facemask	- F_I_O_2_: 0.21–1 - PEEP: 5–8 cmH2O - Continuous gas flow (>30 L/min) + PEEP valve (only CPAP) - PS: 7–10 cmH2O (only NIV)	- The reliable delivery of conditioned gas at the F_I_O_2_ set by the clinician - PEEP-related alveolar recruitment - PS-related inspiratory muscles unload (only NIV) - Tidal volume monitoring (only NIV)	- Skin ulcers - Air leaks and the consequent impairment of high-PEEP delivery - The synchronous PS-related risk of positive P_L_ swings - Poor tolerability, which requires treatment interruptions
	Helmet	- F_I_O_2_: 0.21–1 - PEEP: 10–12 cmH_2_O - Continuous gas flow (>60 L/min) + PEEP valve (only CPAP) for Venturi system devices - PS: 10–12 cmH_2_O (only NIV) - Fastest pressurization time - No need of humidification for minute ventilation above 40 L/min	- The reliable delivery of conditioned gas at the F_I_O_2_ set by the clinician - High PEEP-related alveolar recruitment and homogeneity - PS-related inspiratory muscles unload (only NIV) - Asynchronous PS-related prevention of positive P_L_ swings - Greater patient comfort compared to facemask approach	- No tidal volume measurement - Upper limb edema and a high risk of vasal thrombosis

Abbreviations: CPAP, continuous positive airway pressure; F_I_O_2_, inspired oxygen fraction; NIV, noninvasive ventilation; PEEP, positive end-expiratory pressure; and PL, transpulmonary pressure.

**Table 2:** Monitoring tools and clinical thresholds for the prompt identification of noninvasive respiratory supports failure in patients with AHRF/ARDS.

Parameter	Monitoring Tool	Clinical Threshold Associated with Failure	Limitations
SpO_2_/FiO_2_	Pulse oximetry	<120 and/or worsening trend	Underestimation of clinical severity for low P_a_CO_2_ levels
P_a_O_2_/F_I_O_2_	Arterial blood gas analysis	<150–200 and/or worsening trend	Intermittent
Respiratory rate	Clinical examination	>25–30 and/or not decreasing	Low correlation with effort
Expired tidal volume	Mechanical ventilator	>9–9.5 mL/kg PBW	Not feasible during HFOT and helmet-NIV/CPAP
ΔP_ES_	Esophageal balloon catheter	Absolute value > 10–15 cmH_2_O Reduction of less than 10 cmH_2_O after two hours of NIV	Needs some expertise
ROX index	(SpO_2_/F_I_O_2_)/Respiratory rate	<2.85 at 2 h of HFOT initiation <3.47 at 6 h of HFOT initiation <3.85 at 12 h of HFOT initiation	Validated for HFOT and NIV
HACOR scale	Heart rate, acidosis, stream of consciousness, oxygenation, and respiratory rate. An updated version takes into account some baseline variables as pneumonia, cardiogenic pulmonary edema, pulmonary ARDS, immunosuppression, septic shock, and the SOFA score	>5 points at 1 h of NIV initiation. A HACOR score of ≤7, 7.5–10.5, 11–14, and >14 were updated to be classified at low, moderate, high, or a very high probability of NIV failure	Intermittent, time consuming, and validated only for NIV

Abbreviations: F_I_O_2_, inspired oxygen fraction; HFOT, high-flow oxygen therapy; NIV, noninvasive ventilation; P_a_O_2_, partial arterial O_2_ pressure; PBW, predicted body weight; P_a_CO_2_, partial arterial CO_2_ pressure; ΔP_ES_, delta esophageal pressure; and SpO_2_, peripheral O_2_-saturation.

**Table 3:** Safe limits of ventilatory variables during controlled and assisted invasive mechanical ventilation.

Ventilatory Variables	Initial Setting	Safe Limits
Controlled ventilation		
V_T_	6 mL/kg IBW, targeting a ΔP < 15 cmH_2_O and P_PLAT_ < 28–30 cmH_2_O	Up to 8 mL/kg of IBW if P_PLAT_ and ΔP remain within a safe limit and if 4 × ΔP + RR is reduced
ΔP	/	<15 cmH_2_O, unless more than 4 breaths per minute are needed to maintain isocapnia for each cmH_2_O of ΔP reduction (4 × ΔP + RR is increased)
P_PLAT_	/	<28–30 cmH_2_O
PEEP	High or low PEEP set according to the recruitability profile (e.g., CT-scan, R/I ratio, EIT, esophageal manometry)	P_PLAT_ should be kept within the safe limit; hemodynamic instability should be avoided and treated; and high PEEP in non-recruitable patients is discouraged
RR	Set to maintain PaCO_2_ and pH in the desired range	Check for the presence of PEEPi and set I:E accordingly, and variations of RR should be made in relation with Vt, not to increase 4 × ΔP + RR
Assisted ventilation		
P_0.1_	/	1–4 cmH_2_O
ΔP_OCC_	/	10–15 cmH_2_O
P_PLAT_	/	<28–30 cmH_2_O

Abbreviations: IBW, ideal body weight; ΔP, driving pressure; ΔP_OCC,_ pressure drop during occluded breath; P_PLAT_, plateau pressure; PEEP, positive end-expiratory pressure; RR, respiratory rate; and Vt, tidal volume.

## Data Availability

Not applicable.

## References

[1] Bellani G., Laffey J.G., Pham T., Fan E., Brochard L., Esteban A., Gattinoni L., van Haren F., Larsson A., McAuley D.F. (2016). Epidemiology, patterns of care, and mortality for patients with acute respiratory distress syndrome in intensive care units in 50 countries. JAMA.

[2] Fanelli V., Ranieri V.M. (2015). Mechanisms and clinical consequences of acute lung injury. Ann. Am. Thorac. Soc..

[3] Thompson B.T., Chambers R.C., Liu K.D. (2017). Acute respiratory distress syndrome. N. Engl. J. Med..

[4] Gattinoni L., Pesenti A. (2005). The concept of “baby lung”. Intensive Care Med..

[5] Ashbaugh D.G., Bigelow D.B., Petty T.L., Levine B.E. (1967). Acute respiratory distress in adults. Lancet.

[6] Force A.D.T., Ranieri V.M., Rubenfeld G.D., Thompson B.T., Ferguson N.D., Caldwell E., Fan E., Camporota L., Slutsky A.S. (2012). Acute respiratory distress syndrome: The berlin definition. JAMA.

[7] Ranieri V.M., Tonetti T., Navalesi P., Nava S., Antonelli M., Pesenti A., Grasselli G., Grieco D.L., Menga L.S., Pisani L. (2022). High-flow nasal oxygen for severe hypoxemia: Oxygenation response and outcome in patients with COVID-19. Am. J. Respir. Crit. Care Med..

[8] Rochwerg B., Brochard L., Elliott M.W., Hess D., Hill N.S., Nava S., Navalesi P., Antonelli M., Brozek J., Conti G. (2017). Official ers/ats clinical practice guidelines: Noninvasive ventilation for acute respiratory failure. Eur. Respir. J..

[9] Fan E., Del Sorbo L., Goligher E.C., Hodgson C.L., Munshi L., Walkey A.J., Adhikari N.K.J., Amato M.B.P., Branson R., Brower R.G. (2017). An official american thoracic society/european society of intensive care medicine/society of critical care medicine clinical practice guideline: Mechanical ventilation in adult patients with acute respiratory distress syndrome. Am. J. Respir. Crit Care Med..

[10] Bos L.D.J., Ware L.B. (2022). Acute respiratory distress syndrome: Causes, pathophysiology, and phenotypes. Lancet.

[11] Maddali M.V., Churpek M., Pham T., Rezoagli E., Zhuo H., Zhao W., He J., Delucchi K.L., Wang C., Wickersham N. (2022). Validation and utility of ards subphenotypes identified by machine-learning models using clinical data: An observational, multicohort, retrospective analysis. Lancet Respir. Med..

[12] Matthay M.A., Arabi Y.M., Siegel E.R., Ware L.B., Bos L.D.J., Sinha P., Beitler J.R., Wick K.D., Curley M.A.Q., Constantin J.M. (2020). Phenotypes and personalized medicine in the acute respiratory distress syndrome. Intensive Care Med..

[13] Calfee C.S., Delucchi K., Parsons P.E., Thompson B.T., Ware L.B., Matthay M.A., Network N.A. (2014). Subphenotypes in acute respiratory distress syndrome: Latent class analysis of data from two randomised controlled trials. Lancet Respir. Med..

[14] Gattinoni L., Pesenti D. (1987). Ards: The non-homogeneous lung; facts and hypothesis. Intensive Crit. Care Dig..

[15] Gattinoni L., Pesenti A., Avalli L., Rossi F., Bombino M. (1987). Pressure-volume curve of total respiratory system in acute respiratory failure. Computed tomographic scan study. Am. Rev. Respir. Dis..

[16] Gattinoni L., Pesenti A., Baglioni S., Vitale G., Rivolta M., Pelosi P. (1988). Inflammatory pulmonary edema and positive end-expiratory pressure: Correlations between imaging and physiologic studies. J. Thorac. Imaging.

[17] Gattinoni L., D’Andrea L., Pelosi P., Vitale G., Pesenti A., Fumagalli R. (1993). Regional effects and mechanism of positive end-expiratory pressure in early adult respiratory distress syndrome. JAMA.

[18] Langer M., Mascheroni D., Marcolin R., Gattinoni L. (1988). The prone position in ards patients. A clinical study. Chest.

[19] Gattinoni L., Pelosi P., Vitale G., Pesenti A., D’Andrea L., Mascheroni D. (1991). Body position changes redistribute lung computed-tomographic density in patients with acute respiratory failure. Anesthesiology.

[20] Gattinoni L., Marini J.J., Pesenti A., Quintel M., Mancebo J., Brochard L. (2016). The “baby lung” became an adult. Intensive Care Med..

[21] Bone R.C. (1993). The ards lung. New insights from computed tomography. JAMA.

[22] Yoshida T., Grieco D.L., Brochard L., Fujino Y. (2020). Patient self-inflicted lung injury and positive end-expiratory pressure for safe spontaneous breathing. Curr. Opin. Crit. Care.

[23] Grieco D.L., Menga L.S., Eleuteri D., Antonelli M. (2019). Patient self-inflicted lung injury: Implications for acute hypoxemic respiratory failure and ards patients on non-invasive support. Minerva Anestesiol..

[24] Carteaux G., Parfait M., Combet M., Haudebourg A.F., Tuffet S., Mekontso Dessap A. (2021). Patient-self inflicted lung injury: A practical review. J. Clin. Med..

[25] Carteaux G., Millan-Guilarte T., De Prost N., Razazi K., Abid S., Thille A.W., Schortgen F., Brochard L., Brun-Buisson C., Mekontso Dessap A. (2016). Failure of noninvasive ventilation for de novo acute hypoxemic respiratory failure: Role of tidal volume. Crit. Care Med..

[26] Frat J.P., Ragot S., Coudroy R., Constantin J.M., Girault C., Prat G., Boulain T., Demoule A., Ricard J.D., Razazi K. (2018). Predictors of intubation in patients with acute hypoxemic respiratory failure treated with a noninvasive oxygenation strategy. Crit. Care Med..

[27] Cressoni M., Chiumello D., Algieri I., Brioni M., Chiurazzi C., Colombo A., Colombo A., Crimella F., Guanziroli M., Tomic I. (2017). Opening pressures and atelectrauma in acute respiratory distress syndrome. Intensive Care Med..

[28] Chiumello D., Chierichetti M., Tallarini F., Cozzi P., Cressoni M., Polli F., Colombo R., Castelli A., Gattinoni L. (2008). Effect of a heated humidifier during continuous positive airway pressure delivered by a helmet. Crit. Care.

[29] Yoshida T., Torsani V., Gomes S., De Santis R.R., Beraldo M.A., Costa E.L., Tucci M.R., Zin W.A., Kavanagh B.P., Amato M.B. (2013). Spontaneous effort causes occult pendelluft during mechanical ventilation. Am. J. Respir. Crit. Care Med..

[30] Yoshida T., Roldan R., Beraldo M.A., Torsani V., Gomes S., De Santis R.R., Costa E.L., Tucci M.R., Lima R.G., Kavanagh B.P. (2016). Spontaneous effort during mechanical ventilation: Maximal injury with less positive end-expiratory pressure. Crit. Care Med..

[31] Slutsky A.S., Ranieri V.M. (2013). Ventilator-induced lung injury. N. Engl. J. Med..

[32] Kumar A., Pontoppidan H., Falke K.J., Wilson R.S., Laver M.B. (1973). Pulmonary barotrauma during mechanical ventilation. Crit. Care Med..

[33] Slutsky A.S. (1999). Lung injury caused by mechanical ventilation. Chest.

[34] Grieco D.L., Maggiore S.M., Roca O., Spinelli E., Patel B.K., Thille A.W., Barbas C.S.V., de Acilu M.G., Cutuli S.L., Bongiovanni F. (2021). Non-invasive ventilatory support and high-flow nasal oxygen as first-line treatment of acute hypoxemic respiratory failure and ards. Intensive Care Med..

[35] Luyt C.E., Bouadma L., Morris A.C., Dhanani J.A., Kollef M., Lipman J., Martin-Loeches I., Nseir S., Ranzani O.T., Roquilly A. (2020). Pulmonary infections complicating ards. Intensive Care Med..

[36] Antonelli M., Conti G., Rocco M., Bufi M., De Blasi R.A., Vivino G., Gasparetto A., Meduri G.U. (1998). A comparison of noninvasive positive-pressure ventilation and conventional mechanical ventilation in patients with acute respiratory failure. N. Engl. J. Med..

[37] Putensen C., Mutz N.J., Putensen-Himmer G., Zinserling J. (1999). Spontaneous breathing during ventilatory support improves ventilation-perfusion distributions in patients with acute respiratory distress syndrome. Am. J. Respir. Crit. Care Med..

[38] Chanques G., Constantin J.M., Devlin J.W., Ely E.W., Fraser G.L., Gelinas C., Girard T.D., Guerin C., Jabaudon M., Jaber S. (2020). Analgesia and sedation in patients with ards. Intensive Care Med..

[39] Alhazzani W., Belley-Cote E., Moller M.H., Angus D.C., Papazian L., Arabi Y.M., Citerio G., Connolly B., Denehy L., Fox-Robichaud A. (2020). Neuromuscular blockade in patients with ards: A rapid practice guideline. Intensive Care Med..

[40] Bellani G., Laffey J.G., Pham T., Madotto F., Fan E., Brochard L., Esteban A., Gattinoni L., Bumbasirevic V., Piquilloud L. (2017). Noninvasive ventilation of patients with acute respiratory distress syndrome. Insights from the lung safe study. Am. J. Respir. Crit. Care Med..

[41] Demoule A., Girou E., Richard J.C., Taille S., Brochard L. (2006). Benefits and risks of success or failure of noninvasive ventilation. Intensive Care Med..

[42] Brochard L., Slutsky A., Pesenti A. (2017). Mechanical ventilation to minimize progression of lung injury in acute respiratory failure. Am. J. Respir. Crit. Care Med..

[43] Cesarano M., Grieco D.L., Michi T., Munshi L., Menga L.S., Delle Cese L., Ruggiero E., Rosa T., Natalini D., Sklar M.C. (2022). Helmet noninvasive support for acute hypoxemic respiratory failure: Rationale, mechanism of action and bedside application. Ann. Intensive Care.

[44] Cutuli S.L., Grieco D.L., Menga L.S., De Pascale G., Antonelli M. (2021). Noninvasive ventilation and high-flow oxygen therapy for severe community-acquired pneumonia. Curr. Opin. Infect. Dis..

[45] Rochwerg B., Einav S., Chaudhuri D., Mancebo J., Mauri T., Helviz Y., Goligher E.C., Jaber S., Ricard J.D., Rittayamai N. (2020). The role for high flow nasal cannula as a respiratory support strategy in adults: A clinical practice guideline. Intensive Care Med..

[46] Natalini D., Grieco D.L., Santantonio M.T., Mincione L., Toni F., Anzellotti G.M., Eleuteri D., Di Giannatale P., Antonelli M., Maggiore S.M. (2019). Physiological effects of high-flow oxygen in tracheostomized patients. Ann. Intensive Care.

[47] Chiumello D., Pelosi P., Carlesso E., Severgnini P., Aspesi M., Gamberoni C., Antonelli M., Conti G., Chiaranda M., Gattinoni L. (2003). Noninvasive positive pressure ventilation delivered by helmet vs. Standard face mask. Intensive Care Med..

[48] Taccone P., Hess D., Caironi P., Bigatello L.M. (2004). Continuous positive airway pressure delivered with a “helmet”: Effects on carbon dioxide rebreathing. Crit. Care Med..

[49] Mojoli F., Iotti G.A., Gerletti M., Lucarini C., Braschi A. (2008). Carbon dioxide rebreathing during non-invasive ventilation delivered by helmet: A bench study. Intensive Care Med..

[50] MacIntyre N.R. (2019). Physiologic effects of noninvasive ventilation. Respir. Care.

[51] L’Her E., Deye N., Lellouche F., Taille S., Demoule A., Fraticelli A., Mancebo J., Brochard L. (2005). Physiologic effects of noninvasive ventilation during acute lung injury. Am. J. Respir. Crit. Care Med..

[52] Mehta S., Hill N.S. (2001). Noninvasive ventilation. Am. J. Respir. Crit. Care Med..

[53] Cabrini L., Landoni G., Oriani A., Plumari V.P., Nobile L., Greco M., Pasin L., Beretta L., Zangrillo A. (2015). Noninvasive ventilation and survival in acute care settings: A comprehensive systematic review and metaanalysis of randomized controlled trials. Crit. Care Med..

[54] Luce J.M. (1984). The cardiovascular effects of mechanical ventilation and positive end-expiratory pressure. JAMA.

[55] Luecke T., Pelosi P. (2005). Clinical review: Positive end-expiratory pressure and cardiac output. Crit. Care.

[56] Ueta K., Tomita T., Uchiyama A., Ohta N., Iguchi N., Goto Y., Fujino Y. (2013). Influence of humidification on comfort during noninvasive ventilation with a helmet. Respir. Care.

[57] Lucchini A., Bambi S., Elli S., Bruno M., Dallari R., Puccio P., Villa S., Rona R., Fumagalli R., Bellani G. (2019). Water content of delivered gases during helmet continuous positive airway pressure in healthy subjects. Acta Biomed..

[58] Lenique F., Habis M., Lofaso F., Dubois-Rande J.L., Harf A., Brochard L. (1997). Ventilatory and hemodynamic effects of continuous positive airway pressure in left heart failure. Am. J. Respir. Crit. Care Med..

[59] Rittayamai N., Beloncle F., Goligher E.C., Chen L., Mancebo J., Richard J.M., Brochard L. (2017). Effect of inspiratory synchronization during pressure-controlled ventilation on lung distension and inspiratory effort. Ann. Intensive Care.

[60] Richard J.C., Lyazidi A., Akoumianaki E., Mortaza S., Cordioli R.L., Lefebvre J.C., Rey N., Piquilloud L., Sferrazza Papa G.F., Mercat A. (2013). Potentially harmful effects of inspiratory synchronization during pressure preset ventilation. Intensive Care Med..

[61] Grieco D.L., Menga L.S., Raggi V., Bongiovanni F., Anzellotti G.M., Tanzarella E.S., Bocci M.G., Mercurio G., Dell’Anna A.M., Eleuteri D. (2020). Physiological comparison of high-flow nasal cannula and helmet noninvasive ventilation in acute hypoxemic respiratory failure. Am. J. Respir. Crit. Care Med..

[62] Frat J.P., Thille A.W., Mercat A., Girault C., Ragot S., Perbet S., Prat G., Boulain T., Morawiec E., Cottereau A. (2015). High-flow oxygen through nasal cannula in acute hypoxemic respiratory failure. N. Engl. J. Med..

[63] Ospina-Tascon G.A., Calderon-Tapia L.E., Garcia A.F., Zarama V., Gomez-Alvarez F., Alvarez-Saa T., Pardo-Otalvaro S., Bautista-Rincon D.F., Vargas M.P., Aldana-Diaz J.L. (2021). Effect of high-flow oxygen therapy vs conventional oxygen therapy on invasive mechanical ventilation and clinical recovery in patients with severe COVID-19: A randomized clinical trial. JAMA.

[64] Ferreyro B.L., Angriman F., Munshi L., Del Sorbo L., Ferguson N.D., Rochwerg B., Ryu M.J., Saskin R., Wunsch H., da Costa B.R. (2020). Association of noninvasive oxygenation strategies with all-cause mortality in adults with acute hypoxemic respiratory failure: A systematic review and meta-analysis. JAMA.

[65] Perkins G.D., Ji C., Connolly B.A., Couper K., Lall R., Baillie J.K., Bradley J.M., Dark P., Dave C., De Soyza A. (2022). Effect of noninvasive respiratory strategies on intubation or mortality among patients with acute hypoxemic respiratory failure and COVID-19: The recovery-rs randomized clinical trial. JAMA.

[66] Grieco D.L., Menga L.S., Cesarano M., Rosa T., Spadaro S., Bitondo M.M., Montomoli J., Falo G., Tonetti T., Cutuli S.L. (2021). Effect of helmet noninvasive ventilation vs high-flow nasal oxygen on days free of respiratory support in patients with COVID-19 and moderate to severe hypoxemic respiratory failure: The henivot randomized clinical trial. JAMA.

[67] Arabi Y.M., Aldekhyl S., Al Qahtani S., Al-Dorzi H.M., Abdukahil S.A., Al Harbi M.K., Al Qasim E., Kharaba A., Albrahim T., Alshahrani M.S. (2022). Effect of helmet noninvasive ventilation vs usual respiratory support on mortality among patients with acute hypoxemic respiratory failure due to COVID-19: The helmet-covid randomized clinical trial. JAMA.

[68] Radermacher P., Maggiore S.M., Mercat A. (2017). Fifty years of research in ards. Gas exchange in acute respiratory distress syndrome. Am. J. Respir. Crit. Care Med..

[69] Morais C.C.A., Koyama Y., Yoshida T., Plens G.M., Gomes S., Lima C.A.S., Ramos O.P.S., Pereira S.M., Kawaguchi N., Yamamoto H. (2018). High positive end-expiratory pressure renders spontaneous effort noninjurious. Am. J. Respir. Crit. Care Med..

[70] Lindqvist J., van den Berg M., van der Pijl R., Hooijman P.E., Beishuizen A., Elshof J., de Waard M., Girbes A., Spoelstra-de Man A., Shi Z.H. (2018). Positive end-expiratory pressure ventilation induces longitudinal atrophy in diaphragm fibers. Am. J. Respir. Crit. Care Med..

[71] Sklienka P., Frelich M., Bursa F. (2023). Patient self-inflicted lung injury-a narrative review of pathophysiology, early recognition, and management options. J. Pers. Med..

[72] Brochard L., Harf A., Lorino H., Lemaire F. (1989). Inspiratory pressure support prevents diaphragmatic fatigue during weaning from mechanical ventilation. Am. Rev. Respir. Dis..

[73] Brochard L., Isabey D., Piquet J., Amaro P., Mancebo J., Messadi A.A., Brun-Buisson C., Rauss A., Lemaire F., Harf A. (1990). Reversal of acute exacerbations of chronic obstructive lung disease by inspiratory assistance with a face mask. N. Engl. J. Med..

[74] Coudroy R., Chen L., Pham T., Piraino T., Telias I., Brochard L. (2019). Acute respiratory distress syndrome: Respiratory monitoring and pulmonary physiology. Semin. Respir. Crit. Care Med..

[75] Dangers L., Montlahuc C., Kouatchet A., Jaber S., Meziani F., Perbet S., Similowski T., Resche-Rigon M., Azoulay E., Demoule A. (2018). Dyspnoea in patients receiving noninvasive ventilation for acute respiratory failure: Prevalence, risk factors and prognostic impact: A prospective observational study. Eur. Respir. J..

[76] Grieco D.L., Menga L.S., Cesarano M., Spadaro S., Bitondo M.M., Berardi C., Rosa T., Bongiovanni F., Maggiore S.M., Antonelli M. (2022). Phenotypes of patients with COVID-19 who have a positive clinical response to helmet noninvasive ventilation. Am. J. Respir. Crit. Care Med..

[77] Menga L.S., Delle Cese L., Rosa T., Cesarano M., Scarascia R., Michi T., Biasucci D.G., Ruggiero E., dell’Anna A.M., Cutuli S.L. (2023). Respective effects of helmet pressure support, continuous positive airway pressure and nasal high-flow in hypoxemic respiratory failure: A randomized crossover clinical trial. Am. J. Respir. Crit. Care Med..

[78] Kangelaris K.N., Ware L.B., Wang C.Y., Janz D.R., Zhuo H., Matthay M.A., Calfee C.S. (2016). Timing of intubation and clinical outcomes in adults with acute respiratory distress syndrome. Crit. Care Med..

[79] Duan J., Han X., Bai L., Zhou L., Huang S. (2017). Assessment of heart rate, acidosis, consciousness, oxygenation, and respiratory rate to predict noninvasive ventilation failure in hypoxemic patients. Intensive Care Med..

[80] Spinelli E., Mauri T., Beitler J.R., Pesenti A., Brodie D. (2020). Respiratory drive in the acute respiratory distress syndrome: Pathophysiology, monitoring, and therapeutic interventions. Intensive Care Med..

[81] Tonelli R., Fantini R., Tabbi L., Castaniere I., Pisani L., Pellegrino M.R., Della Casa G., D’Amico R., Girardis M., Nava S. (2020). Early inspiratory effort assessment by esophageal manometry predicts noninvasive ventilation outcome in de novo respiratory failure. A pilot study. Am. J. Respir. Crit. Care Med..

[82] Roca O., Caralt B., Messika J., Samper M., Sztrymf B., Hernandez G., Garcia-de-Acilu M., Frat J.P., Masclans J.R., Ricard J.D. (2019). An index combining respiratory rate and oxygenation to predict outcome of nasal high-flow therapy. Am. J. Respir. Crit. Care Med..

[83] Duan J., Yang J., Jiang L., Bai L., Hu W., Shu W., Wang K., Yang F. (2022). Prediction of noninvasive ventilation failure using the rox index in patients with de novo acute respiratory failure. Ann. Intensive Care.

[84] Duan J., Chen L., Liu X., Bozbay S., Liu Y., Wang K., Esquinas A.M., Shu W., Yang F., He D. (2022). An updated hacor score for predicting the failure of noninvasive ventilation: A multicenter prospective observational study. Crit. Care.

[85] Lassen H.C. (1953). A preliminary report on the 1952 epidemic of poliomyelitis in copenhagen with special reference to the treatment of acute respiratory insufficiency. Lancet.

[86] Papazian L., Forel J.M., Gacouin A., Penot-Ragon C., Perrin G., Loundou A., Jaber S., Arnal J.M., Perez D., Seghboyan J.M. (2010). Neuromuscular blockers in early acute respiratory distress syndrome. N. Engl. J. Med..

[87] National Heart L., Blood Institute P.C.T.N., Moss M., Huang D.T., Brower R.G., Ferguson N.D., Ginde A.A., Gong M.N., Grissom C.K., Gundel S. (2019). Early neuromuscular blockade in the acute respiratory distress syndrome. N. Engl. J. Med..

[88] Dreyfuss D., Soler P., Basset G., Saumon G. (1988). High inflation pressure pulmonary edema. Respective effects of high airway pressure, high tidal volume, and positive end-expiratory pressure. Am. Rev. Respir. Dis..

[89] Amato M.B., Barbas C.S., Medeiros D.M., Magaldi R.B., Schettino G.P., Lorenzi-Filho G., Kairalla R.A., Deheinzelin D., Munoz C., Oliveira R. (1998). Effect of a protective-ventilation strategy on mortality in the acute respiratory distress syndrome. N. Engl. J. Med..

[90] Acute Respiratory Distress Syndrome N., Brower R.G., Matthay M.A., Morris A., Schoenfeld D., Thompson B.T., Wheeler A. (2000). Ventilation with lower tidal volumes as compared with traditional tidal volumes for acute lung injury and the acute respiratory distress syndrome. N. Engl. J. Med..

[91] Terragni P.P., Rosboch G., Tealdi A., Corno E., Menaldo E., Davini O., Gandini G., Herrmann P., Mascia L., Quintel M. (2007). Tidal hyperinflation during low tidal volume ventilation in acute respiratory distress syndrome. Am. J. Respir. Crit. Care Med..

[92] Chiumello D., Carlesso E., Cadringher P., Caironi P., Valenza F., Polli F., Tallarini F., Cozzi P., Cressoni M., Colombo A. (2008). Lung stress and strain during mechanical ventilation for acute respiratory distress syndrome. Am. J. Respir. Crit. Care Med..

[93] Amato M.B., Meade M.O., Slutsky A.S., Brochard L., Costa E.L., Schoenfeld D.A., Stewart T.E., Briel M., Talmor D., Mercat A. (2015). Driving pressure and survival in the acute respiratory distress syndrome. N. Engl. J. Med..

[94] Gattinoni L., Tonetti T., Cressoni M., Cadringher P., Herrmann P., Moerer O., Protti A., Gotti M., Chiurazzi C., Carlesso E. (2016). Ventilator-related causes of lung injury: The mechanical power. Intensive Care Med..

[95] Camporota L., Busana M., Marini J.J., Gattinoni L. (2021). The 4dprr index and mechanical power: A step ahead or four steps backward?. Am. J. Respir. Crit. Care Med..

[96] Costa E.L.V., Slutsky A.S., Brochard L.J., Brower R., Serpa-Neto A., Cavalcanti A.B., Mercat A., Meade M., Morais C.C.A., Goligher E. (2021). Ventilatory variables and mechanical power in patients with acute respiratory distress syndrome. Am. J. Respir. Crit. Care Med..

[97] Huhle R., Serpa Neto A., Schultz M.J., Gama de Abreu M. (2018). Is mechanical power the final word on ventilator-induced lung injury?-no. Ann. Transl. Med..

[98] Maggiore S.M., Jonson B., Richard J.C., Jaber S., Lemaire F., Brochard L. (2001). Alveolar derecruitment at decremental positive end-expiratory pressure levels in acute lung injury: Comparison with the lower inflection point, oxygenation, and compliance. Am. J. Respir. Crit. Care Med..

[99] Richard J.C., Maggiore S.M., Jonson B., Mancebo J., Lemaire F., Brochard L. (2001). Influence of tidal volume on alveolar recruitment. Respective role of peep and a recruitment maneuver. Am. J. Respir. Crit. Care Med..

[100] Bellani G., Guerra L., Musch G., Zanella A., Patroniti N., Mauri T., Messa C., Pesenti A. (2011). Lung regional metabolic activity and gas volume changes induced by tidal ventilation in patients with acute lung injury. Am. J. Respir. Crit. Care Med..

[101] Gattinoni L., Caironi P., Cressoni M., Chiumello D., Ranieri V.M., Quintel M., Russo S., Patroniti N., Cornejo R., Bugedo G. (2006). Lung recruitment in patients with the acute respiratory distress syndrome. N. Engl. J. Med..

[102] Grieco D.L., Chen L., Brochard L. (2017). Transpulmonary pressure: Importance and limits. Ann. Transl. Med..

[103] Cavalcanti A.B., Suzumura E.A., Laranjeira L.N., Paisani D.M., Damiani L.P., Guimaraes H.P., Romano E.R., Regenga M.M., Taniguchi L.N.T., Writing Group for the Alveolar Recruitment for Acute Respiratory Distress Syndrome Trial (ART) Investigators (2017). Effect of lung recruitment and titrated positive end-expiratory pressure (peep) vs low peep on mortality in patients with acute respiratory distress syndrome: A randomized clinical trial. JAMA.

[104] Brower R.G., Lanken P.N., MacIntyre N., Matthay M.A., Morris A., Ancukiewicz M., Schoenfeld D., Thompson B.T., National Heart L., Blood Institute A.C.T.N. (2004). Higher versus lower positive end-expiratory pressures in patients with the acute respiratory distress syndrome. N. Engl. J. Med..

[105] Meade M.O., Cook D.J., Guyatt G.H., Slutsky A.S., Arabi Y.M., Cooper D.J., Davies A.R., Hand L.E., Zhou Q., Thabane L. (2008). Ventilation strategy using low tidal volumes, recruitment maneuvers, and high positive end-expiratory pressure for acute lung injury and acute respiratory distress syndrome: A randomized controlled trial. JAMA.

[106] Mercat A., Richard J.C., Vielle B., Jaber S., Osman D., Diehl J.L., Lefrant J.Y., Prat G., Richecoeur J., Nieszkowska A. (2008). Positive end-expiratory pressure setting in adults with acute lung injury and acute respiratory distress syndrome: A randomized controlled trial. JAMA.

[107] Kacmarek R.M., Villar J., Sulemanji D., Montiel R., Ferrando C., Blanco J., Koh Y., Soler J.A., Martinez D., Hernandez M. (2016). Open lung approach for the acute respiratory distress syndrome: A pilot, randomized controlled trial. Crit. Care Med..

[108] Briel M., Meade M., Mercat A., Brower R.G., Talmor D., Walter S.D., Slutsky A.S., Pullenayegum E., Zhou Q., Cook D. (2010). Higher vs lower positive end-expiratory pressure in patients with acute lung injury and acute respiratory distress syndrome: Systematic review and meta-analysis. JAMA.

[109] Constantin J.M., Jabaudon M., Lefrant J.Y., Jaber S., Quenot J.P., Langeron O., Ferrandiere M., Grelon F., Seguin P., Ichai C. (2019). Personalised mechanical ventilation tailored to lung morphology versus low positive end-expiratory pressure for patients with acute respiratory distress syndrome in france (the live study): A multicentre, single-blind, randomised controlled trial. Lancet Respir. Med..

[110] Chen L., Del Sorbo L., Grieco D.L., Junhasavasdikul D., Rittayamai N., Soliman I., Sklar M.C., Rauseo M., Ferguson N.D., Fan E. (2020). Potential for lung recruitment estimated by the recruitment-to-inflation ratio in acute respiratory distress syndrome. A clinical trial. Am. J. Respir. Crit. Care Med..

[111] Chen L., Del Sorbo L., Grieco D.L., Shklar O., Junhasavasdikul D., Telias I., Fan E., Brochard L. (2018). Airway closure in acute respiratory distress syndrome: An underestimated and misinterpreted phenomenon. Am. J. Respir. Crit. Care Med..

[112] Costa E.L., Borges J.B., Melo A., Suarez-Sipmann F., Toufen C., Bohm S.H., Amato M.B. (2009). Bedside estimation of recruitable alveolar collapse and hyperdistension by electrical impedance tomography. Intensive Care Med..

[113] Yoshida T., Piraino T., Lima C.A.S., Kavanagh B.P., Amato M.B.P., Brochard L. (2019). Regional ventilation displayed by electrical impedance tomography as an incentive to decrease positive end-expiratory pressure. Am. J. Respir. Crit. Care Med..

[114] Yoshida T., Amato M.B.P., Grieco D.L., Chen L., Lima C.A.S., Roldan R., Morais C.C.A., Gomes S., Costa E.L.V., Cardoso P.F.G. (2018). Esophageal manometry and regional transpulmonary pressure in lung injury. Am. J. Respir. Crit. Care Med..

[115] Sarge T., Baedorf-Kassis E., Banner-Goodspeed V., Novack V., Loring S.H., Gong M.N., Cook D., Talmor D., Beitler J.R., Group E.P.-S. (2021). Effect of esophageal pressure-guided positive end-expiratory pressure on survival from acute respiratory distress syndrome: A risk-based and mechanistic reanalysis of the epvent-2 trial. Am. J. Respir. Crit. Care Med..

[116] De Jong A., Verzilli D., Jaber S. (2019). Ards in obese patients: Specificities and management. Crit. Care.

[117] Chen L., Grieco D.L., Beloncle F., Chen G.Q., Tiribelli N., Madotto F., Fredes S., Lu C., Antonelli M., Mercat A. (2022). Partition of respiratory mechanics in patients with acute respiratory distress syndrome and association with outcome: A multicentre clinical study. Intensive Care Med..

[118] Suarez-Sipmann F., Bohm S.H., Tusman G. (2014). Volumetric capnography: The time has come. Curr. Opin. Crit. Care.

[119] Kallet R.H., Zhuo H., Liu K.D., Calfee C.S., Matthay M.A., National Heart L., Blood Institute A.N.I. (2014). The association between physiologic dead-space fraction and mortality in subjects with ards enrolled in a prospective multi-center clinical trial. Respir. Care.

[120] Fengmei G., Jin C., Songqiao L., Congshan Y., Yi Y. (2012). Dead space fraction changes during peep titration following lung recruitment in patients with ards. Respir. Care.

[121] Pham T., Telias I., Piraino T., Yoshida T., Brochard L.J. (2018). Asynchrony consequences and management. Crit. Care Clin..

[122] de Vries H., Jonkman A., Shi Z.H., Spoelstra-de Man A., Heunks L. (2018). Assessing breathing effort in mechanical ventilation: Physiology and clinical implications. Ann. Transl. Med..

[123] Carson S.S., Kress J.P., Rodgers J.E., Vinayak A., Campbell-Bright S., Levitt J., Bourdet S., Ivanova A., Henderson A.G., Pohlman A. (2006). A randomized trial of intermittent lorazepam versus propofol with daily interruption in mechanically ventilated patients. Crit. Care Med..

[124] Vaschetto R., Cammarota G., Colombo D., Longhini F., Grossi F., Giovanniello A., Della Corte F., Navalesi P. (2014). Effects of propofol on patient-ventilator synchrony and interaction during pressure support ventilation and neurally adjusted ventilatory assist. Crit. Care Med..

[125] Bouillon T., Bruhn J., Roepcke H., Hoeft A. (2003). Opioid-induced respiratory depression is associated with increased tidal volume variability. Eur. J. Anaesthesiol..

[126] Pattinson K.T. (2008). Opioids and the control of respiration. Br. J. Anaesth..

[127] Belleville J.P., Ward D.S., Bloor B.C., Maze M. (1992). Effects of intravenous dexmedetomidine in humans. I. Sedation, ventilation, and metabolic rate. Anesthesiology.

[128] Telias I., Abbott M., Brochard L. (2021). Monitoring respiratory drive and effort during mechanical ventilation. J. Transl. Crit. Care Med..

[129] Whitelaw W.A., Derenne J.P., Milic-Emili J. (1975). Occlusion pressure as a measure of respiratory center output in conscious man. Respir. Physiol..

[130] Bertoni M., Telias I., Urner M., Long M., Del Sorbo L., Fan E., Sinderby C., Beck J., Liu L., Qiu H. (2019). A novel non-invasive method to detect excessively high respiratory effort and dynamic transpulmonary driving pressure during mechanical ventilation. Crit. Care.

[131] Bellani G., Grassi A., Sosio S., Foti G. (2019). Plateau and driving pressure in the presence of spontaneous breathing. Intensive Care Med..

[132] Guerin C., Reignier J., Richard J.C., Beuret P., Gacouin A., Boulain T., Mercier E., Badet M., Mercat A., Baudin O. (2013). Prone positioning in severe acute respiratory distress syndrome. N. Engl. J. Med..

[133] Munshi L., Del Sorbo L., Adhikari N.K.J., Hodgson C.L., Wunsch H., Meade M.O., Uleryk E., Mancebo J., Pesenti A., Ranieri V.M. (2017). Prone position for acute respiratory distress syndrome. A systematic review and meta-analysis. Ann. Am. Thorac. Soc..

[134] Papazian L., Munshi L., Guerin C. (2022). Prone position in mechanically ventilated patients. Intensive Care Med..

[135] Sardo S., Osawa E.A., Finco G., Gomes Galas F.R.B., de Almeida J.P., Cutuli S.L., Frassanito C., Landoni G., Hajjar L.A. (2018). Nitric oxide in cardiac surgery: A meta-analysis of randomized controlled trials. J. Cardiothorac. Vasc. Anesth..

[136] Gebistorf F., Karam O., Wetterslev J., Afshari A. (2016). Inhaled nitric oxide for acute respiratory distress syndrome (ards) in children and adults. Cochrane Database Syst. Rev..

